# A comparison of right ventricular volumes in children and adults with repaired tetralogy of Fallot - including and excluding right ventricular trabeculations

**DOI:** 10.1186/1532-429X-18-S1-P169

**Published:** 2016-01-27

**Authors:** James D Enos, Jacob Hartz, Rachel Quinn, Lasya Gaur, Russell Cross, Laura Olivieri

**Affiliations:** Cardiology, Children's National Health System, Washington, DC USA

## Background

Tetralogy of Fallot (ToF) repair often involves placing a transannular patch to augment the right ventricular outflow tract, resulting in right heart dilation and failure over time. Right ventricular (RV) volume measurement by cardiac magnetic resonance imaging (CMR) is the gold standard to guide timing of pulmonary valve replacement in this population - often at an indexed right ventricular end-diastolic volume (RVEDVi) > 160 ml/m^2^. The RV volume is measured by tracing the endocardial border on a stack of SSFP cine images, with most approaches including RV trabeculations in the blood pool. Semi-automated, threshold-based software (Mass-K, Medis, Leiden, The Netherlands) allows for volume measurements that exclude trabeculations from the RV with the aim of improved accuracy of the true blood pool volume. Threshold-based analysis has yielded significantly different RV volumes and ejection fraction in the adult population. This study compares the impact of threshold-based analysis of RV volumes in children and adults following ToF repair.

## Methods

**Figure 1:**
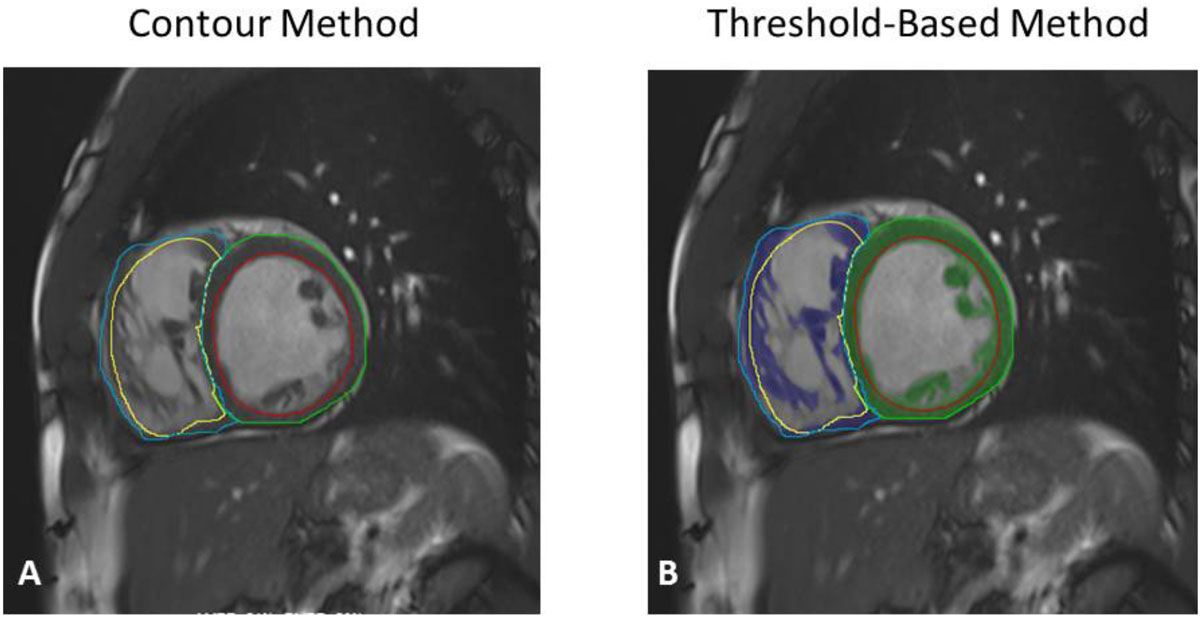
**Two SSFP images in the short axis demonstrating (A) the standard, contour-based method of RV volume analysis with trabeculations included in the blood pool, and (B) a threshold-based method of RV volume analysis with trabeculations excluded from the blood pool**.

Thirty-nine patients with repaired ToF underwent clinically indicated CMR including twenty-one pediatric patients ≤ 18 years. Indexed RV end-diastolic and end-systolic volume measurements (RVEDVi and RVESVi) were repeated for independent comparison using the standard contour method and the threshold-based method (Figure 1). Summary statistics were obtained for the two methods via the paired-samples Student's t-test using STATA/IC 13.1 (Table 1).

**Table 1:** Table 1

	Pediatrics			Adults		
	Contour	Threshold	p-value	Contour	Threshold	p-value
RVEDVi, ml/m^2^	144 ± 13	113 ± 12	<0.001	148 ± 20	111 ± 18	<0.001
RVESVi, ml/m^2^	73 ± 11	51 ± 7	<0.001	84 ± 14	58 ± 12	<0.001
RVEF	50% ± 7	56% ± 6	<0.001	44% ± 4	48% ± 5	<0.001

## Results

In the pediatric age group, the mean RVEDVi using the contour method was 144 ml/m^2^, and 113 ml/m^2^ using threshold-based analysis (mean difference 31 ml/m^2^, p-value < 0.001). Similarly, the pediatric RVESVi using the contour method was 73 ml/m^2^, and 51 ml/m^2^ using threshold-based analysis (mean difference 22 ml/m^2^, p-value < 0.001). The pediatric RVEF was 50 percent using the contour method and 56 percent using threshold-based analysis (mean difference 6 percent, p-value < 0.001). In the pediatric subgroup, seven patients had an RVEDVi greater than 160 ml/m^2^ using the contour method, while none had an RVEDVi greater than 160 ml/m^2^ using the threshold-based method. The adult subgroup had similar significant differences in RVEDVi, RVESVi, and RVEF - consistent with previously reported data. In the adult group, six patients had an RVEDVi greater than 160 ml/m^2^ using the contour method, while only one had an RVEDVi greater than 160 ml/m^2^ using the threshold method.

## Conclusions

Threshold-based and contour-based analysis of RV volumes using CMR data yields differing RV volume measurements in both children and adults with repaired ToF. This analysis demonstrates that care must be taken prior to incorporating alternative endocardial contour techniques into clinical decision-making. Incorporating these techniques into clinical practice may help refine indications and timing of pulmonary valve replacement by accounting for RV trabeculations, which was previously difficult using traditional contour methods.

